# The evolution of household forgone essential care and its determinants during the COVID-19 pandemic in Nigeria: A longitudinal analysis

**DOI:** 10.1371/journal.pone.0296301

**Published:** 2024-04-02

**Authors:** Adelakun Odunyemi, Hamid Sohrabi, Khurshid Alam

**Affiliations:** 1 Murdoch Business School, Murdoch University, Perth, Western Australia; 2 Hospitals’ Management Board, Alagbaka Estate, Akure, Ondo State, Nigeria; 3 Centre for Healthy Ageing, Murdoch University, Perth, Western Australia; APHRC: African Population and Health Research Center, ETHIOPIA

## Abstract

Apart from the morbidity and mortality, the Coronavirus disease 2019 (COVID-19) pandemic has increased the predisposition of households in Nigeria to forgone care, thereby increasing their vulnerability to adverse health consequences. Since there is no previous study in Nigeria on the evolution of pandemic-related forgone care and its drivers, our study assess the evolution of the problem using descriptive and nationally representative panel data analyses. We found about a 30% prevalence of forgone care during the lockdown, which declined progressively afterwards, dropping by 69.50 percentage points between April 2020 and April 2022. This decline produced a surge in households needing care from about 35.00% in the early pandemic to greater than 50%, beginning in early 2021. The forgone care was primarily due to financial hindrances, movement restrictions, and supply-side disruptions. Household socioeconomic factors such as income loss had 2.74 [95%CI: 1.45–5.17] times higher odds of forgone care, job loss, food insecurity, and poverty were 87% (OR: 1.87 [95%CI: 1.25–2.79]), 60% (OR: 1.60 [95%CI: 1.12–2.31]) and 76% (OR: 1.76 [95%CI: 1.12–2.75]) more likely to predispose households to forgone care, respectively. Also, geographical location, such as the South-South zone, induced 1.98 [95%CI: 1.09–3.58] times higher odds of forgone care than North-Central. A married female household head increased the odds by 6.07 [95%CI: 1.72–21.47] times compared with an unmarried female head. However, having a married household head, social assistance, and North-East or North-West zone compared with North-Central increased the chance of accessing care by 69% (OR 0.31 [95%CI: 0.16–0.59]), 59%,(OR 0.41 [95%CI: 0.21–0.77]), 72% (OR 0.28 [95%CI: 0.15–0.53]) and 64% (OR 0.36 [95%CI: 0.20–0.65]), respectively. Non-communicable diseases, disability, old age, large household size and rural-urban location did not affect the forgone care. Our study highlights the need to strengthen Nigeria’s health system, create policies to promote healthcare accessibility and prepare the country for future pandemic challenges.

## Introduction

In recent history, only a few events have been able to produce as much global socioeconomic disruption and devastation as the Coronavirus disease 2019 (COVID-19) caused by the SARS-CoV-2 virus infection. Apart from the disease burden, the COVID-19 pandemic has worsened the pre-existing inequalities in health access and detrimental health outcomes in most countries [1]. Before the pandemic, more than half the world’s population had no access to essential health services such as medicine, maternal and child health (MCH), adult health, preventive care (child vaccination and family planning), and emergency care [2]. The pandemic resulted in a one-third reduction in healthcare utilisation, with a greater decline among individuals with non-COVID-19 illnesses [3]. For example, with obvious detrimental sequelae, a decrease in medication adherence was found among patients with chronic diseases, including diabetes, hypertension, and cardiovascular diseases during the pandemic [4]. The consequences of severe disruptions in family planning and contraception services in 9% of countries, as reported by the World Health Organization (WHO), are also predictable [5]. Moreover, there was a worldwide reduction or delay in routine vaccination and MCH services [6, 7], leading to adverse health outcomes, such as an increase in maternal and perinatal mortalities, ruptured ectopic pregnancies, and preterm deliveries [8]. Although lower cases and morbidity from COVID-19 were reported in Sub-Saharan Africa (SSA), including Nigeria [9], the pandemic caused devastating socioeconomic effects in the region [10].

Nigeria was leading in the prevalence of pandemic-induced forgone care and its adverse consequences in SSA [11, 12]. For example, 84% of people with noncommunicable diseases (NCDs) in Nigeria experienced deterioration in their conditions during the pandemic because they were unable to access their medicine [13]. Direct and indirect effects of the pandemic were propelling the high prevalence of forgone care. The growing burden of the COVID-19 infection and the restriction measures by the government exerted consequential effects on healthcare access [10, 14]. This saw many households forgoing essential care with attendant serious health outcomes [15, 16]. These impacts occurred amidst a poorly-funded, deficient, and fragile healthcare system, leading to a considerably disruption in access to essential healthcare services and widespread socioeconomic and health problems in Nigeria [17, 18]. The socioeconomic impacts which potentially contributed to the prevalence of forgone care include pandemic-induced income and job losses, and resulting heightened household food insecurity [10, 19]. This has brought about reduction in household consumption and aggravated poverty [20, 21].Worsening the situation was the principal dependence of the populace on out-of-pocket (OOP) payments for healthcare access [22]. Thus, in the SSA region, Nigeria suffered the highest rate of forgone care with its detrimental outcomes during the pandemic [12].

Considering the severe implications of forgone care on health outcomes, studies on the prevalence of forgone care in Nigeria are pertinent. However, only a handful of literature has attempted to provide an overview of healthcare service disruption or forgone care in Nigeria [18, 23]. Kakietek et al. (2022) estimated the prevalence of foregone care and the various reported reasons for forgoing care during the early COVID-19 pandemic in 39 low-and medium-income countries (LMICs), including Nigeria [11]. However, they only captured a time-snap during the lockdown and failed to examine household factors driving forgone care. Also, a group of World Bank researchers analysed the impacts of COVID-19 on human capital, livelihoods and welfare in Nigeria and snapshot access to medical treatment between April 2020 and January 2021 [17]. Unfortunately, these studies are not comprehensive enough to capture the evolution in the prevalence of forgone care and its drivers in Nigeria. Therefore, our study aims to assess the evolving prevalence and the drivers of forgone care during the COVID-19 pandemic in Nigeria, using panel data spanning the three years of the pandemic.

### Conceptual framework

**Fig 1:**
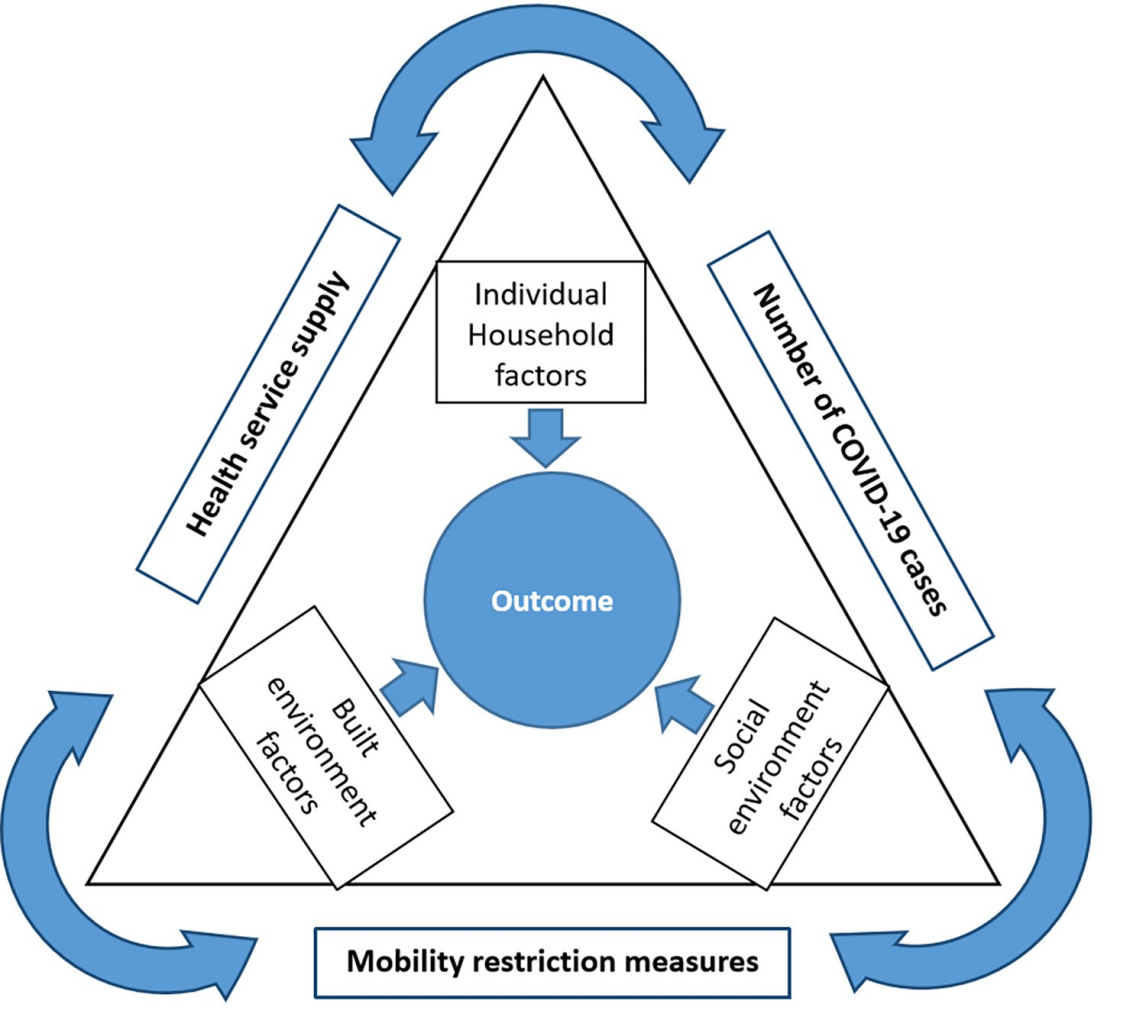
Conceptual model of determinants of healthcare utilisation during COVID-19 pandemic. *Notes*: The conceptual framework shows three extrinsic factors (outside the triangle) and three intrinsic factors (inside the triangle). The extrinsic factors are those induced by the pandemic and operate outside the immediate environment of a household. They include the number of COVID-19 cases, government mobility restriction policy and disruption in the supply of health services. They exist in a vicious circle. The Intrinsic factors are those that operate within a household even before the pandemic. The *Individual household factors* were adapted from the three classical factors of Andersen’s Behavioural Model of Health Service Utilization (BMHSU) and included predisposing, enabling (or disabling) and need factors [24, 27]. The predisposing factors are socio-demographic and health beliefs of the household head, such as age, gender, marital status, and education. The enabling (or disenabling) factors are resources required for accessing care (e.g., household finance, number of working adults, health insurance, and adult-equivalent household size). The need factors include household actual or diagnosed or perceived health status of its members (e.g., presence of chronic diseases or elderly). The remaining two intrinsic factors are elements in the physical environment of the household, based on the work of Ryvicker [26]. They include social environmental factors or social capital (e.g., social conflicts, social support and community health) and built environmental factors (e.g., transportation and communication systems).

To explore the factors driving household forgone care during the pandemic, we developed a triangular conceptual framework for the determinants of healthcare utilisation documented in the literature (Fig 1). There are two categories of factors: Extrinsic (outside the triangle) and Intrinsic factors (inside the triangle). There are three extrinsic factors, which are outside the immediate environment of a household, that interplayed during the pandemic. They include the number of COVID-19 cases, government mobility restriction policy and supply of health services. These three factors existed in a vicious circle, one influencing the other. We examined these extrinsic factors descriptively (i.e. reasons for forgoing care). Three categories of intrinsic factors are outplayed within a household, making it vulnerable to forgone care. Individual household factors were adapted from the three classical factors of Andersen’s Behavioural Model of Health Service Utilization (BMHSU): predisposing, enabling (or disabling) and need factors [24]. Although the popular BMHSU primarily focuses on individual factors, the initial model was designed with the household in mind [24, 25]. The proponent of the model recognised that the care received by an individual is a function of the demographic and socioeconomic composition of the household [24]. Thus, we adopted this model with a more restrictive definition to classify our household factors.

Since household decision-making primarily lies with the household head, the predisposing factors are socio-demographic and health beliefs of the household head, such as age, gender, marital status, and education (including health literacy). The enabling (or disenabling) factors are resources necessary for accessing care (e.g., household finance, number of working adults, health insurance, and adult-equivalent household size). The need factors include household actual or diagnosed or perceived health status of the household members (e.g., presence of chronic diseases or elderly). The remaining two intrinsic factors are elements in the milieu where a household is located. This part of our model was inspired by the work of Ryvicker (2018) [26]. They include social environmental factors or social capital (e.g., social conflicts, social support and community health) and built environmental factors (e.g., transportation and communication systems). Both extrinsic and intrinsic factors interact in several ways. For example, restriction measures can affect household income and transportation. All these factors contribute cumulatively to determining household access to care during the pandemic. Our model is designed to understand factors that push household forgone care during the pandemic.

## Method

### Data description

We conducted a longitudinal study using three sets of nationally representative panel data collected between 2019 and 2022 by the Nigeria National Bureau of Statistics (NBS) in collaboration with the World Bank as part of the Living Standard Measurement Studies (LSMS) [28]. The first set is the pre-COVID-19 face-to-face General Household Survey Panel (GHSP), collected during the post-harvest season between January and February 2019. The GHSP survey formed a sampling frame from which subsequent COVID-19 surveys used in this study were drawn. It provided helpful information on the characteristics of the households before the pandemic. The GHSP comprises 5,000 households randomly sampled across 500 enumeration areas (EAs), giving it a national spread. Other information about the GHSP is detailed elsewhere [29]. The second and third sets are the first (NLPS-1) and second (NLPS-2) phases of the COVID-19 national longitudinal phone survey (NLPS) conducted monthly from April 2020 to April 2021, and bi-monthly from November 2021 to November 2022, respectively. Households in both NLPS-1 and NLPS-2 were randomly sampled from the GHSP. Data were collected at a high-frequency rate (monthly or bi-monthly) to examine the near real-time impacts of the pandemic and restriction measures on households’ economy, food security, employment, and access to essential services, including healthcare. For NLPS-1, a nationally representative sample of approximately 1,800 successfully interviewed households was targeted. A 60% final contact-plus-response rate was targeted to minimise non-contact and non-response biases common in telephone surveys. An excess pool of 3,000 households was selected from 4,934, which were households that provided at least one telephone contact during the 2019 survey, representing approximately 99% of the original GHPS households. About 90% (4,440 households) of these households provided at least one household member’ number, while the remaining 10% only provided a reference person’s number. The final sample in the baseline (round 1) consisted of 1,950 households, after accounting for those that were successfully contacted and fully interviewed (69% and 94%, respectively) (S1 Table). These were households contacted in consequent rounds. The information collected from these households retrospectively extended to mid-March 2020. We used only seven rounds of NLPS-1, shown in S1 Table, because they were the only ones containing health access data.

Considering the difficulty encountered during NLPS-1 in contacting households which provided a reference person’s contact, only those that supplied a member’s number were contacted in NLPS-2. All 4,440 households that provided at least one member’s phone number in GHSP (wave 4) were contacted in NLPS-2. This included 2,701 of 3,000 households that provided a household member’s number in the baseline of NLPS-1. With a 65% response rate in the NLPS-1 baseline sample, about 2,900 households were expected to complete the NLPS-2 interview. In round 1, 64.8% (2,922 households) of the 4,440 contacted were fully interviewed (S2 Table). These 2,922 households were the final sample contacted in subsequent rounds of NLPS-2. Our study used four rounds of NLPS-2 which contains health access data (S2 Table).

A balanced sampling method, using the cube method, was adopted to ensure the selected households in NLPS-1 and NLPS-2 retained the properties of the original GHSP frame [30, 31]. This technique reduced the variance between the randomly selected sample and the original frame across the chosen covariates. The sample was balanced across the following covariates: state, urban-rural location, household size, sex and education of the household head, and household ownership of assets, including mobile phones.

**Fig 2:**
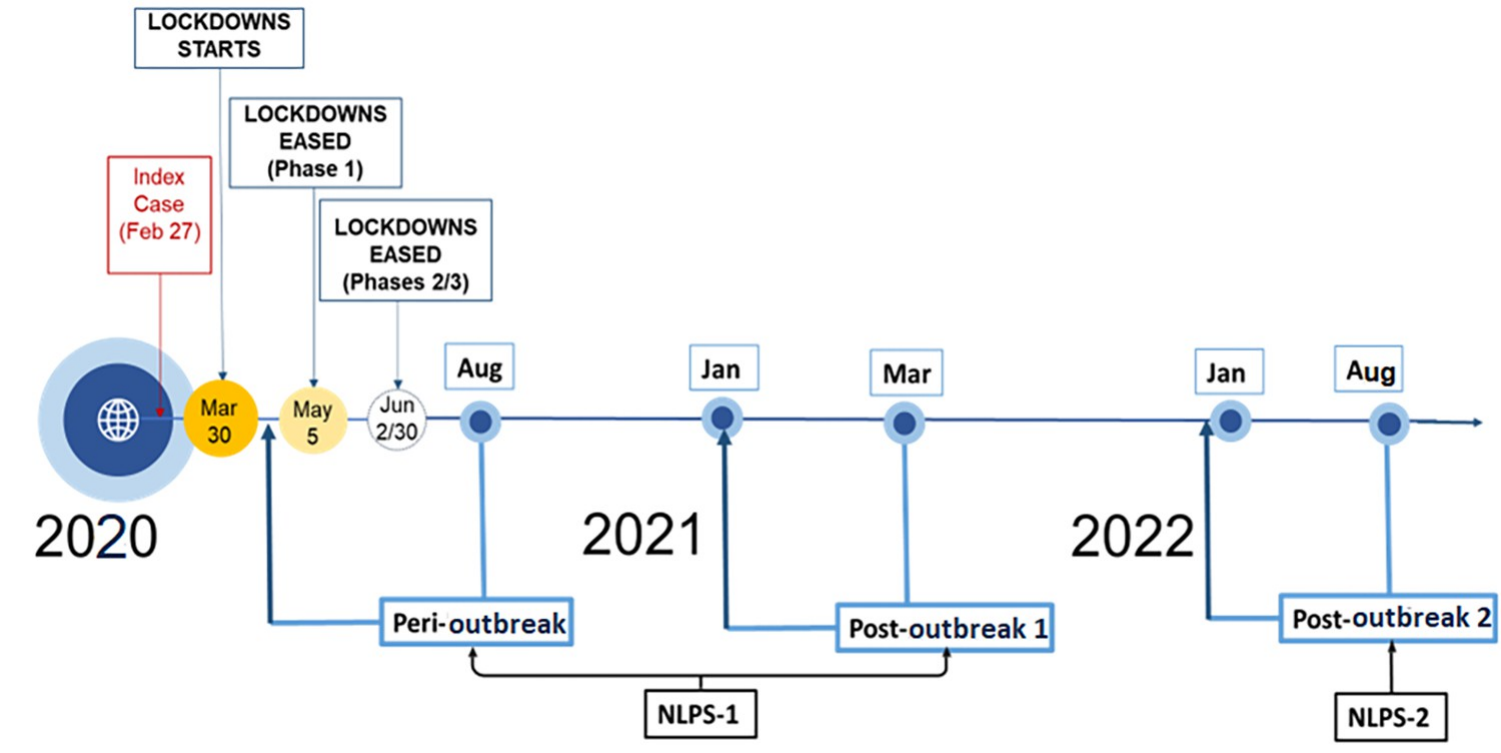
The timeline of COVID-19 pandemic and national longitudinal phone surveys(NLPS) data collection in Nigeria. Notes: *Peri-outbreak* represents a period from April to August 2020. *Post-outbreak 1 and 2* represent a period from January 2021 to December 2021, with some gaps from April to October 2021 and January to August 2022, respectively. NLPS-1 and NLPS-2 represent Nigeria COVID-19 National Longitudinal Phone Surveys phase 1 and phase 2 respectively.

We analysed data collected over three years, from 2020 to 2022, and divided them, for descriptive purpose, into three distinct non-continuous periods: the *peri-outbreak* (2020), *post-outbreak 1* (2021), and *post-outbreak 2* (2022) periods. The *peri-outbreak* period includes data collected in 2020, specifically the NLPS-1, rounds 1 to 4, collected between April and August 2020. This period captures the lockdown period in April-May 2020 to the peak of the pandemic’s first wave in June 2020 (see S1 Fig, Lower Panel) and immediately after (Fig 2). There is no health utilisation data from September to December 2020. The *post-outbreak 1* period covers data collected from January 2021 to December 2021, with a gap of seven months between April and October 2021. Most of the third wave of the infection occurred during this interval between NLPS-1 and NPLS-2. The *post-outbreak 1* period includes the last three rounds of NLPS-1 (rounds 9 to 11) and part of the first round of NLPS-2, collected between November and December 2021 (S1 and S2 Tables). This period mainly telescopes the peak of the second wave in January 2021 (see S1 Fig, Lower Panel) and immediately after. The *post-outbreak 2* period covers 2022 and includes the remaining part of round 1 and rounds 3 to 5 of NLPS-2. Round 2 of NLPS-2 has no health access data. This *post-outbreak 2* period mainly spans from the peak of the third wave in January 2022 to the fourth wave, which began in June 2022 (see S1 Fig, Lower Panel). Except the baseline round of NLPS-1 which used a 4-week recall, all rounds used a seven-day recall period for data collection. The three-point timeline we used provides an opportunity to compare forgone care during the peaks of the pandemic’s waves (S1 Fig, Lower Panel) and between similar periods during the three years of the pandemic in Nigeria, accounting for seasonal variations.

### Variable measures

#### Outcome variable

Forgone care is our outcome variable for this study. A household is considered to have forgone care when they reported needing care but could not access it. We measured forgone care using healthcare access questions in our surveys. The surveys asked two nested questions inquiring about household access to healthcare services: (1) whether the respondents or any member of their households needed medical care during the recall period, and (2) whether they could access the services they needed. These two questions required a no/yes answer. From these two questions, we distinguished three important groups, frequently inseparable in other studies: (1) those that needed healthcare, (2) those able to access needed care, and (3) those unable to access needed care (forgone care). The first group is crucial in understanding the pressure on the health system. The second group reflects the level of access to care and forms a suitable comparator for the third group, our outcome of interest.

#### Explanatory variables

We examine the variability of the above outcomes (forgone care) with time. The survey also asked why people could not assess care in the form of an open-ended question. We matched each care forgone with those reasons given and assessed the trend over time. We categorised the reasons into five as follows: (1) Financial hindrances (lack of money or high cost); (2) Fear of COVID-19; (3) Supply-side disruptions (no medical personnel, unavailable medical supplies, turned away or refused treatment by the facility, health facility closed, and health facility too far); (4) Mobility restrictions (movement restrictions and lack of transportation); (5) Other reasons (the person got well, prefer home remedies, not a serious illness, etc.).

### Statistical analysis

We used descriptive statistics to assess the distribution and prevalence of forgone care, expressing categorical variables as counts or percentages. The prevalence of forgone care is the proportion of households who reported needing care but could not access the care. We reported our findings with their 95% confidence intervals (95% CIs).

To further understand the dynamics of forgone care during the pandemic, we estimated the pull prevalence across the rounds and data collection timeline. We performed a time-trend analysis of the prevalence of forgone medical care, comparing it with the trend in COVID-19 cases, deaths and Oxford Stringency Index (OSI), using data obtained from *Our World in Data* [32]. The OSI is the aggregated strictness scores of governmental COVID-19 policy measures across time developed by the University of Oxford [33].

To explore the intrinsic determinants of household vulnerability to forgone care during the pandemic, we constructed a panel between all the eleven rounds of NLPS used in this study and employed random effects estimator, confirmed by Hausman’s specification test [34] as the most consistent and effective estimator, as follows:

$$y_{ht}=\beta_{i}x_{iht}+u_{h}+t+\varepsilon_{ht}$$

Where *y_ht_* is the dummy for households with and without forgone care. *x_iht_* represents a vector of household-specific factors that influence forgone care, using available variables corresponding to intrinsic household vulnerability factors in the conceptual framework. We added a time dummy, *t* to correct for variations and seasonality in time. *ε_ht_* is an error term, assuming that the individual effects in the error term are uncorrelated with the explanatory variables. In addition, we used Huber-White robust cluster standard errors [35] at the enumeration area (EA) to correct for heteroskedasticity, assuming similar observable and unobservable socioeconomic shocks and service disruptions applied to households living in the same EA. Because our outcome variable is binary, we used binomial logistic regression to estimate our equation. We also verified the accuracy of our model, considering the quadrature method used by Stata to estimate our random effect logistic regressions.

All statistical analyses were performed using Stata/MP 17 (StataCorp, Texas, USA) for Windows. In our analysis of the evolution of forgone care, we applied survey weights disseminated with the datasets to obtain nationally representative estimates. The NLPS-1 and NLPS-2 survey weights were built on the sampling weights of the parent GHSP survey, using well-established methods detailed elsewhere [30, 31]. These weights were calibrated to address the selection bias of not owning a mobile phone and non-response bias from contacted households.

### Ethics statement

This study used fully anonymised secondary data collected by NBS and repository at the online Word Bank microdata page, accessed from March, 2021 to March 2023. Ethical approval for this research was waived by the Murdoch University Human Research Ethics Committee (project approval reference number 2022/159).

## Result

### Summary statistics of household characteristics and access to care

Table 1 compares household characteristics in the survey rounds in the periods before and after the COVID-19 outbreak. Most household characteristics spanning the four years were essentially unchanged. Majority of households (61.80% [95%CI: 58.04–69.29]) in the sample were in the rural area. Most of the household heads (79.88% [95%CI: 78.74–80.97]) were male, and 74% [95%CI: 72.76–75.20] of them were married. A majority (63.08% [(95%CI: 61.7–64.41]) of the household heads had some form of formal education. Before the outbreak, about 40.11% [95%CI: 38.76–41.48] of the households were below the national poverty line, and 12.99% [95%CI: 12.09–13.95] had persons with NCDs.

**Table 1:** Household sample composition in the rounds of the surveys in the year before (2019) and the three years after the outbreak of Covid-19 in Nigeria (2020–2022).

Household Characteristics^α^	Survey Rounds^β^											
**Household’s geopolitical zone**	**0**	**1**	**2**	**3**	**4**	**9**	**10**	**11**	**13**	**15**	**16**	**17**
North-Central *n (%)*	845 (15.28) [14.02–16.63]	331 (15.28) [13.22–17.59]	299 (15.28) [13.09–17.75]	287 (15.28) [13.07–17.78]	295 (15.28) [13.10–17.175]	276 (15.28) [13.05–17.82]	274 (15.28) [13.06–17.79]	274 (15.28) [13.04–17.83]	758 (15.28) [13.51–17.23]	497 (15.28) [13.39–17.37]	495 (15.28) [13.42–17.35]	219 (15.28) [13.37–17.41]
North-East *n (%)*	825 (11.14) [10.04–12.35]	331 (11.14) [9.45–13.09]	317 (11.14) [9.41–13.15]	321 (11.14) [9.43–13.12]	319 (11.14) [9.41–13.14]	312 (11.15) [9.40–13.17]	312 (11.14) [9.40–13.16]	307 (11.15) [9.37–13.21]	692 (11.14) [9.63–12.85]	484 (11.14) [9.58–12.92]	483 (11.14) [9.57–12.93]	295 (11.14) [9.54–12.97]
North-West *n (%)*	843 (22.78) [21.09–24.57]	308 (22.78) [19.97–25.86]	294 (22.78) [19.92–25.91]	286 (22.78) [19.89–25.95]	278 (22.78) [19.84–26.01]	271 (22.79) [19.84–26.03]	270 (22.78) [19.81–26.05]	265 (22.79) [19.80–26.08]	693 (22.78) [20.29–25.47]	394 (22.78) [20.20–25.58]	389 (22.78) [20.18–25.61]	230 (22.78) [20.08–25.72]
South-East *n (%)*	824 (15.54) [14.30–16.87]	366 (15.54) [13.60–17.71]	330 (15.54) [13.56–17.76]	327 (15.54) [13.50–17.83]	326 (15.54) [13.50–17.83]	308 (15.51) [13.44–17.82]	307 (15.54) [13.43–17.92]	305 (15.51) [13.41–17.86]	777 (15.54) [13.94–17.30]	505 (15.54) [13.89–17.36]	502 (15.54) [13.83–17.43]	252 (15.54) [13.80–17.46]
South-South *n (%)*	815 (17.74) [16.27–19.32]	295 (17.74) [15.35–20.42]	245 (17.74) [15.21–20.59]	238 (17.74) [15.15–20.67]	238 (17.74) [15.17–20.65]	214 (17.75) [15.09–20.76]	218 (17.74) [15.11–20.73]	213 (17.75) [15.07–20.79]	744 (17.74) [15.74–19.94]	422 (17.74) [15.59–20.12]	411 (17.74) [15.59–20.13]	131 17.74 [15.54–20.19]
South-West *n (%)*	824 (17.52) [15.87–19.29]	384 (17.52) [14.93–20.44]	345 (17.52) [14.84–20.57]	349 (17.52) [14.86–20.24]	342 (17.52) [14.86–20.55]	325 (17.52) [14.81–20.61]	327 (17.52) [14.82–20.58]	325 (17.52) [14.83–20.60]	776 (17.52) [15.42–19.84]	579 (17.52) [15.36–19.91]	572 (17.52) [15.31–19.97]	176 (17.52) [15.28–20.00]
**Household’s location**												
Urban *n (%)*	1,592 (31.44) [29.58–33.36]	774 (31.44) [28.57–34.46]	719 (31.44) [28.47–34.56]	710 (31.44) [28.45–34.59]	707 (31.44) [38.44–34.60]	668 (31.43) [28.38–34.62]	668 (31.44) [38.38–34.67]	666 (31.43) [38.35–34.68]	1,533 (31.44) [28.99–33.99]	1,157 (31.44) [28.93–34.06]	1,141 (31.44) [28.87–34.13]	491 (31.44) [28.83–34.17]
Rural *n (%)*	3,384 (68.56) [66.64–70.42]	1,241 (68.56) [65.54–71.43]	1,111 (68.56) [65.44–71.53]	1,098 (68.56) [65.41–71.55]	1,091 (68.56) [65.40–71.56]	1,038 (68.57) [65.35–71.62]	1,040 (68.56) [65.33–71.62]	1,023 (68.57) [65.32–71.65]	2,907 (68.56) [66.01–71.01]	1,724 (68.56) [65.94–71.07]	1,711 (68.56) [65.87–71.13]	812 (68.56) [65.83–71.17]
**Household size (category)**												
Less than seven *n (%)*	3,523 (68.43) [66.54–70.26]	1,259 (60.70) [57.47–63.85]	1,125 (60.06) [56.70–63.32]	1,095 (58.92) [55.52–62.24]	1,077 (58.37) [54.93–61.72]	932 (52.82) [49.31–56.31]	926 (52.64) [49.13–56.13]	911 (51.87) [48.29–55.35]	1,676 (53.28) [50.51–56.03]	1,556 (54.54) [51.63–57.42]	1,489 (53.78) [50.83–56.70]	651 (53.52) [50.50–56.52]
More than seven *n (%)*	1,457 (31.57) [29.74–33.46]	751 (39.30) [36.15–42.53]	705 (39.94) [36.68–43.30]	712 (41.08) [37.76–44.48]	721 (41.63) [38.28–45.07]	774 (47.18) [43.69–50.69]	779 (47.36) [43.87–50.87]	777 (48.17) [44.65–51.71]	1,303 (46.72) [43.97–49.49]	1,113 (45.46) [42.58–48.37]	1,123 (46.22) [43.30–49.17]	652 (46.48) [43.48–59.50]
**NCD-affected households (pre-pandemic)**												
Not NCD-affected *n (%)*	4,333 (87.76) [86.46–88.95]	1,739 (87.47) [85.28–89.37]	1,579 (87.66) [85.37–89.63]	1,562 (87.46) [85.13–89.47]	1,552 (87.70) [85.44–89.65]	1,465 (87.13) [84.78–89.17]	1,468 (87.07) [84.67–89.15]	1,453 (87.22) [84.83–89.28]	3,861 (87.57) [85.64–89.27]	2,505 (87.58) [85.58–89.34]	2,476 (87.96) [86.01–89.67]	1,102 (87.39) [85.28–89.24]
NCD-affected *n (%)*	647 (12.24) [12.05–13.54]	276 (12.53) [10.63–14.72]	251 (12.34) [10.37–14.63]	246 (12.54) [10.53–1.87]	246 (12.30) [10.35–14.56]	241 (12.87) [10.83–15.22]	240 (14.05) [12.48–15.78]	236 (12.78) [10.72–15.17]	579 (12.43) [10.73–14.36]	376 (12.42) [10.66–14.42]	376 (12.04) [10.33–13.99]	201 (12.61) [10.76–14.72]
**Poor Households (pre-pandemic)**												
No *n (%)*	2,980 (61.15) [59.22–63.06]	1,303 (61.24) [57.97–64.41]	1,188 (61.10) [57.70–64.40]	1,173 (61.04) [57.61–64.37]	1,161 (61.06) [57.60–64.41]	1,097 (60.91) [57.40–64.31]	1,100 (60.82) [57.31–64.21]	1,095 (60.67) [57.13–64.10]	2,751 (61.17) [58.41–63.87]	1,880 (61.34) [58.43–64.17]	1,856 (61.34) [58.41–64.20]	821 (61.56) [58.53–64.49]
Yes *n (%)*	1,996 (38.85) [36.94–40.78]	712 (38.76) [35.59–42.03]	642 (38.90) [35.60–42.30]	635 (38.96) [35.63–42.39]	637 (38.94) [35.59–42.40]	609 (39.09) [35.69–42.60]	608 (39.18) [35.79–42.69]	594 (39.33) [35.90–42.87]	1,689 (38.83) [36.13–41.59]	1,001 (38.66) [35.83–41.57]	996 (38.66) [35.80–41.59]	482 (38.44) [35.51–41.47]
**Age of household head (category)**												
18–34 years *n (%)*	820 (17.14) [15.67–18.71]	268 (13.28) [11.25–15.62]	263 (14.14) [12.87–16.61]	263 (14.24) [12.07–16.72]	260 (14.15) [11.99–16.62]	234 (13.29) [11.16–15.76]	231 (13.61) [11.42–16.13]	234 (13.50) [11.32–16.02]	352 (13.19) [11.36–15.25]	299 (12.63) [10.78–14.73]	288 (12.59) [10.72–14.74]	133 (12.51) [10.61–14.69]
35–49 years *n (%)*	1,745 (37.53) [35.59–39.52]	745 (39.05) [35.84–42.35]	681 (37.94) [34.67–41.33]	670 (38.74) [35.40–42.20]	669 (39.55) [36.18–43.02]	642 (39.57) [36.14–43.10]	647 (39.64) [36.22–43.17]	638 (40.06) [36.60–43.62]	1,089 (38.51) [35.82–41.27]	1,008 (38.83) [36.01–41.74]	992 (38.76) [35.91–41.70]	525 (38.96) [36.02–41.99]
50–64 years *n (%)*	1,467 (28.15) [26.44–29.93]	611 (29.85) [26.98–32.89]	564 (30.18) [27.16–33.38]	559 (29.46) [26.49–32.62]	559 28.80) [25.87–31.91]	527 (29.27) [36.14–43.10]	525 (28.89) [25.89–32.09]	516 (29.90) [25.87–32.12]	958 (29.81) [27.40–32.33]	863 (30.60) [28.05–33.27]	852 (30.44) [27.87–33.13]	418 (30.95) [28.32–33.71]
65 years or more *n (%)*	948 (17.18) [15.80–18.65]	359 (18.10) [16.47–19.86]	317 (17.74) [15.30–20.48]	309 (17.55) [15.06–20.36]	305 (17.50) [15.02–20.29]	301 (17.87) [15.34–20.71]	300 (17.86) [15.35–20.69]	294 (17.54) [15.07–20.32]	577 (18.50) [16.53–20.64]	499 (17.95) [15.91–20.17]	480 (18.21) [16.13–20.49	227 (17.57) [15.49–19.87]
**Marital status of household head**												
Married *n (%)*	3,685 (74.96) [73.18–76.66]	1,513 (74.96) [71.99–77.71]	1,379 (75.00) [71.95–77.83]	1,366 (75.06) [71.95–77.93]	1,360 (75.04) [71.92–77.91]	1,300 (74.79) [71.60–77.74]	1,296 (74.64) [71.43–77.60]	1,285 (74.70) [71.47–77.69]	3,324 (74.49) [72.02–76.81]	2,224 (74.53) [71.93–76.96]	2,201 (74.53) [71.92–76.97]	1,052 (74.52) [71.87–77.01]
Unmarried *n (%)*	1,295 (25.04) [23.34–26.82]	501 (25.04) [22.29–28.01]	450 (25.00) [22.17–28.05]	441 (24.94) [22.07–28.93]	437 (24.96) [22.09–28.08]	405 (25.21) [22.26–28.40	411 (25.36) 22.40–28.57]	403 (25.30) [22.32–28.53]	1,114 (25.51) [23.19–27.98]	656 (25.47) [23.04–28.07]	650 (25.47) [23.03–28.08]	250 (25.48) [22.99–28.13]
**Education level of household head**												
None *n (%)*	1,837 (37.84) [35.93–39.78]	571 (37.15) [33.94–40.48]	512 (37.42) [34.08–40.87]	493 (36.85) [33.48–40.35]	487 (37.07) [33.68–40.60]	467 (36.99) [33.57–29.54]	475 (37.27) [33.85–40.83]	463 (37.28) [33.83–40.87]	1,438 (37.76) [35.06–40.54]	786 (37.76) [34.91–40.69]	782 (37.39) [34.54–40.34]	351 (37.13) [34.21–40.15]
Primary *n (%)*	1,219 (23.94) [22.31–25.64]	507 (23.88) [21.26–26.71]	449 (23.37) [20.68–26.31]	453 (23.83) [21.11–26.77]	449 (23.57) [20.86–26.52]	423 (23.66) [20.89–26.69]	417 (23.27) [33.85–40.24]	412 (23.14) [20.37–26.15]	1,129 (23.51) [21.29–25.88]	719 (23.77) [21.45–26.27]	713 (23.84) [21.48–26.38]	313 (24.24) [21.78–26.87]
Secondary *n (%)*	1,095 (22.42) [20.74–24.19]	511 (23.17) [20.51–26.06]	466 (23.44) [20.68–26.45]	464 (23.55) [20.76–26.59]	460 (23.58) [20.80–26.61]	443 (23.56) [20.972–26.65]	437 (23.69) [20.84 26.81]	441 (23.80) [20.94–26.93]	1,056 (22.90) [20.68–25.29]	750 (22.66) [20.34–25.17]	743 (22.96) [20.59–25.50]	329 (22.86) [20.45–25.46]
Tertiary *n (%)*	825 (15.81) [14.46–17.27]	426 (15.80) [13.83–17.99]	403 (15.77) [13.73–18.03]	398 (15.77) [13.71–18.08]	402 (15.78) [13.72–18.08]	373 (15.79) [13.64–18.20]	379 (15.80) [13.68–18.19]	373 (15.78) [13.61–18.21]	817 (15.83) [14.04–17.79]	626 (15.80) [13.99–17.81]	614 (15.81) [13.95–17.86]	310 (15.78) [13.89–17.87]
**Sex of household head**												
Female *n (%)*	1,002 (18.63) [17.15–20.21]	365 (17.87) [15.54–20.47]	338 (18.52) [15.90–20.96]	329 (18.16) [15.72–20.89]	329 (18.34) [15.88–21.08]	302 (19.17) [16.54–22.11]	306 (18.94) [16.35–21.84]	296 (18.45) [15.91–21.29]	544 (18.97) [16.97–21.15]	492 (19.06) [16.97–21.35]	480 (19.45) [1.30–21.78]	217 (19.18) [17.01–21.55]
Male *n (%)*	3,978 (81.37) [79.79–82.85]	1,619 (82.13) [79.53–84.46]	1,487 (81.71) [79.40–84.10]	1,472 (81.84 [79.1–84.28]	1,464 (81.66) [78.92–84.12]	1,402 (80.83) [77.89–83.46]	1,397 (81.06) [78.16–83.65]	1,386 (81.55) [78.71–84.09]	2,432 (81.03) [78.85–83.03]	2,177 (80.94) [78.65–83.03]	2,132 (80.55) [78.22–82.70]	1,086 (80.82) [78.45–82.99]
**Household in need of medical care**												
No *n (%)*	1,475 (30.26) [28.46–32.13]	1,326 (65.73) [62.51–68.80]	1,225 (64.87) [61.52–68.08]	1,107 (60.26) [56.89–63.54]	1,095 (58.76) [55.32–62.14]	788 (46.18) [42.71–49.69]	958 (54.52) [51.00–57.99]	905 (54.05) [50.52–57.54]	1,525 (51.16) [48.40–53.92]	1,503 (54.61) [51.70–57.48]	1,242 (47.21) [44.30–50.14]	1,260 (47.91) [44.93–50.91]
Yes *n (%)*	3,504 (69.74) [67.87–71.54]	651 (34.27) [31.20–37.49]	602 (35.13) [31.92–38.48]	695 (39.54) [36.46–43.11]	702 41.24) [37.86–44.69]	918 (53.82) [50.31–57.29]	745 (45.48) [42.01–49.00]	780 (45.95) [42.46–49.48]	1,433 (48.84) [46.08–51.60]	1,162 (45.39) [42.52–48.30]	1,368 (52.79) [49.86–55.70]	1,318 (52.09) [49.09–55.07]
**Household with forgone medical care**												
No *n (%)*	2,901 (83.36) [81.48–85.09]	493 (74.41) [69.11–79.08]	528 (85.63) [80.70–89.47]	602 (86.39) [82.33–89.63]	637 (89.88) [85.66–92.96]	810 (86.87) [83.18–89.85]	699 (93.53) [90.39–95.69]	745 (96.40) [94.46–97.68]	1,354 (94.08) [91.89–95.70]	1,138 (97.23) [95.03–98.47]	1,337 (93.32) [93.8–97.81]	1,295 (97.54) [95.37–98.70]
Yes *n (%)*	603 (16.64) [14.91–18.52]	157 (25.59) [20.92–30.89]	74 (14.37) [10.53–19.30]	93 (13.61) [10.37–17.68]	65 (10.12) [7.04–14.34]	108 (13.13) [10.15–16.82]	46 (6.47) [4.31–9.61]	35 (3.60) [2.32–5.54]	79 (5.92) [4.30–8.11]	23 (2.77) [1.53–4.97]	31 (3.68) [2.19–6.12]	23 (2.46) [1.30–4.63]

*Notes*: ^**α**^Authors’ calculations based on *weighted* samples of 2018/2019 General Household Survey, Panel (GHSP) wave 4, 2020/2021 Nigeria, COVID-19 National Longitudinal Phone Surveys, phase 1 (NLPS-1), and 2021/2022 Nigeria COVID-19 National Longitudinal Phone Surveys, phase 2 (NLPS-2).

^**β**^The GHSP pre-pandemic round is recorded as 0, and to form a continuous round, rounds 1, 3, 4 and 5 of the NLPS-2 are represented respectively as rounds 13, 15, 16 and 17, respectively.

95% CIs are shown in square brackets.

### The distribution of needed and forgone care

**Fig 3:**
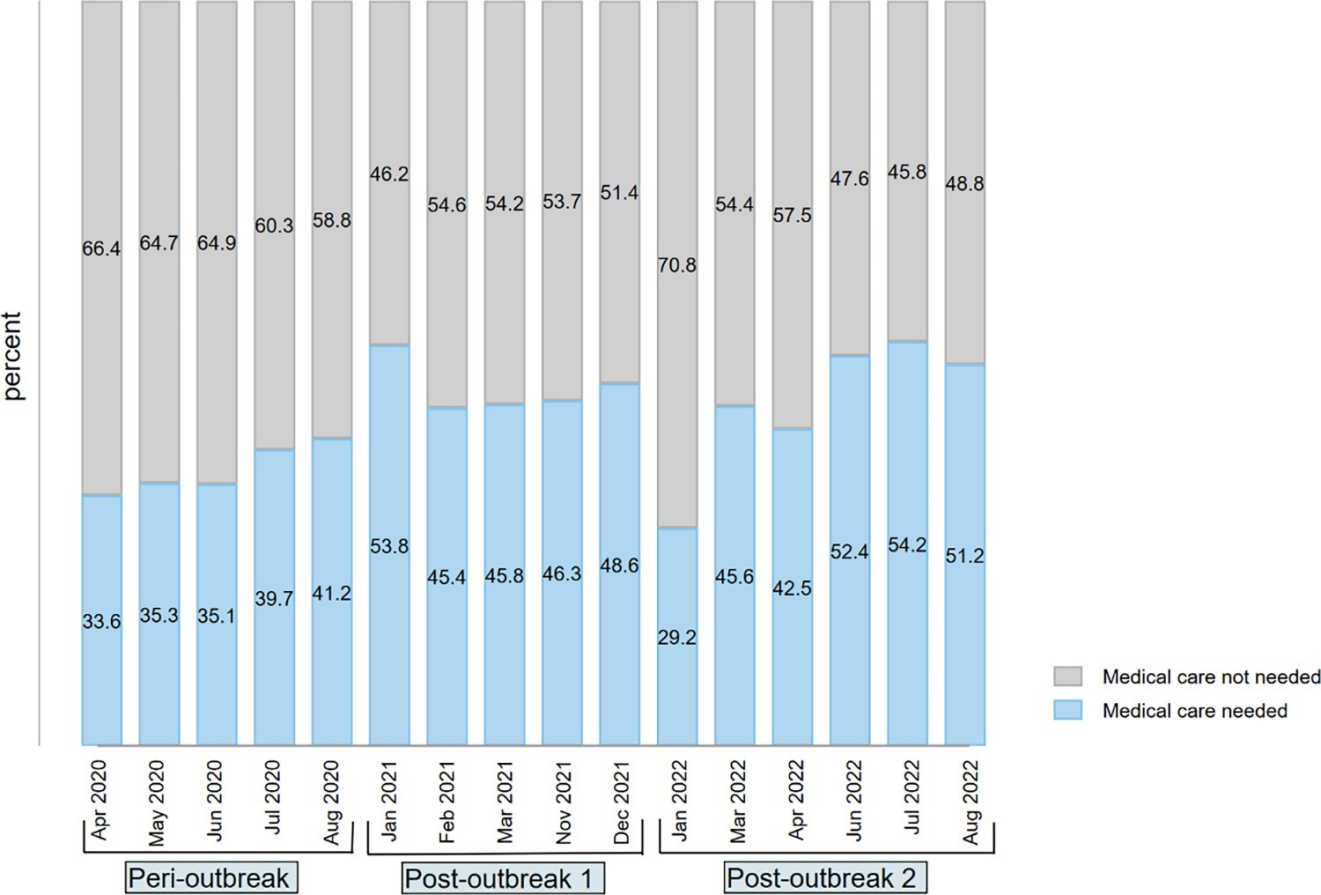
Evolution in the household needs for care during the COVID-19 pandemic in Nigeria (2020–2022). *Notes*: authors’ calculations were based on weighted samples of Nigeria COVID-19 National longitudinal phone surveys (NLPS) 2020/2021 (rounds 1, 2, 3, 4, 9, 10 and 11) and 2021/2022 (rounds 1, 3,4 and 5). *Peri-outbreak* represents a period from April to August 2020. *Post-outbreak 1 and 2* represent a period from January 2021 to December 2021, with some gaps from April to October 2021 and January to August 2022, respectively.

Table 1 also shows heterogeneity in needed and forgone care of households across the years of the surveys. The distribution of households’ need for medical care during the pandemic is shown in Fig 3. The average proportion of households that needed care during the three years of the pandemic was 44.98% [95%CI: 44.00–45.95] (Table 1). The proportion of households requiring care was stable at around 35% during the first three months, an half of the pre-pandemic level (70.38% [95%CI: 69.09–71.63]). However, the number began to rise around July 2020 and, in most cases, was above the peri-outbreak figure, exceeding 50% in early 2021 (post-outbreak 1) and late 2022 (post-outbreak 2). The prevalence was still lower than the pre-pandemic level.

**Fig 4:**
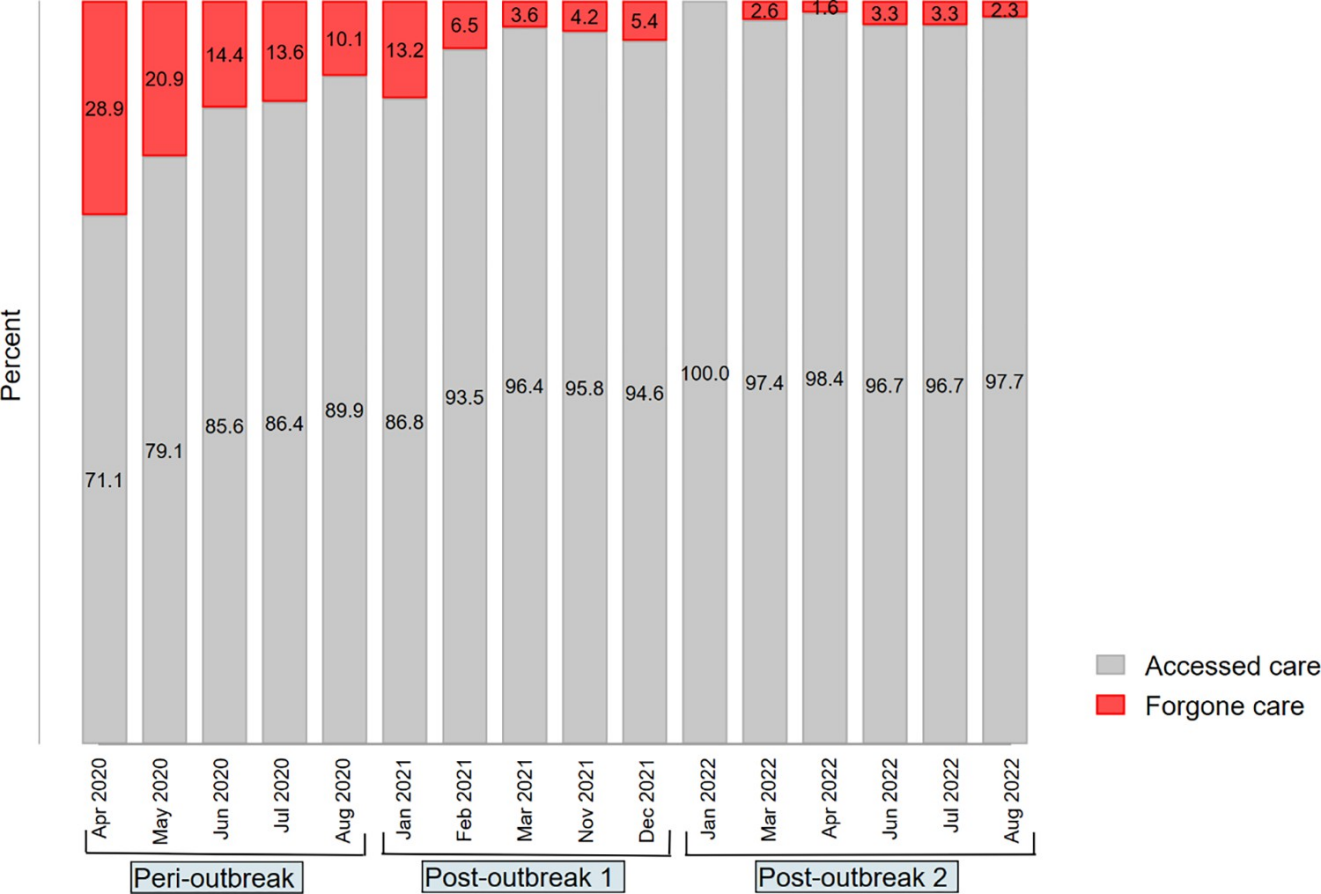
Evolution of household access and forgone care during the COVID-19 pandemic in Nigeria (2020–2022). *Notes*: The table shows the distribution of household access to medical services during the pandemic. With variability, more households could access care than forgone care, even during the early pandemic. The proportion of forgone care was highest in April 2020 and dropped progressively thereafter. Authors’ calculations were based on weighted samples of Nigeria’s COVID-19 National longitudinal phone surveys (NLPS) 2020/2021 (rounds 1, 2, 3, 4, 9, 10 and 11) and 2021/2022 (rounds 1, 3,4 and 5). *Peri-outbreak* represents a period from April to August 2020. *Post-outbreak 1 and 2* depict a period from January 2021 to December 2021, with some gaps from April to October 2021 and January to August 2022, respectively.

Fig 4 shows the proportion of forgone care was about 30.00% in April 2020. However, the proportion began to drop as the pandemic progressed, giving an increase in access by approximately 69.50 percentage points between April 2020 and April 2022. The improvement in medical access was only slightly interrupted around Jan 2021, with a fall of 3.10 percentage points in access, compared with August 2020 and almost at par with June/July 2020 prevalence.

The prevalence of forgone care decreased over time, with the highest prevalence occurring during the lockdown period in April/May 2020 at almost 30.00% (Fig 4). The mean prevalence during the peri-outbreak period was 15.56% [95%CI: 13.49–17.63], representing about 6.3 million [95%CI: 5.50–7.10] households (S3 Table). The prevalence continued to fall during consecutive periods with averages of 7.38% [95% CI: 6.09–8.66] and 2.63% [95% CI: 1.71–3.56] during the "post-outbreak 1" and "post-outbreak 2" periods, respectively. The trend in forgone care appears to respond to the number of COVID-19 cases and deaths and the stringency of measures put in place to control the virus (S1 Fig). The highest prevalence of forgone care occurred during the lockdown period, and the overall trend continued to fall after, with three other lower peaks around July-August 2020, January-February 2021 and December 2021- January 2022. These appear to follow the volume and spikes in the number of COVID-19 cases and deaths, though not proportionately than the stringency index (S1 Fig).

### The pattern of distribution of forgone care across different essential services

**Fig 5:**
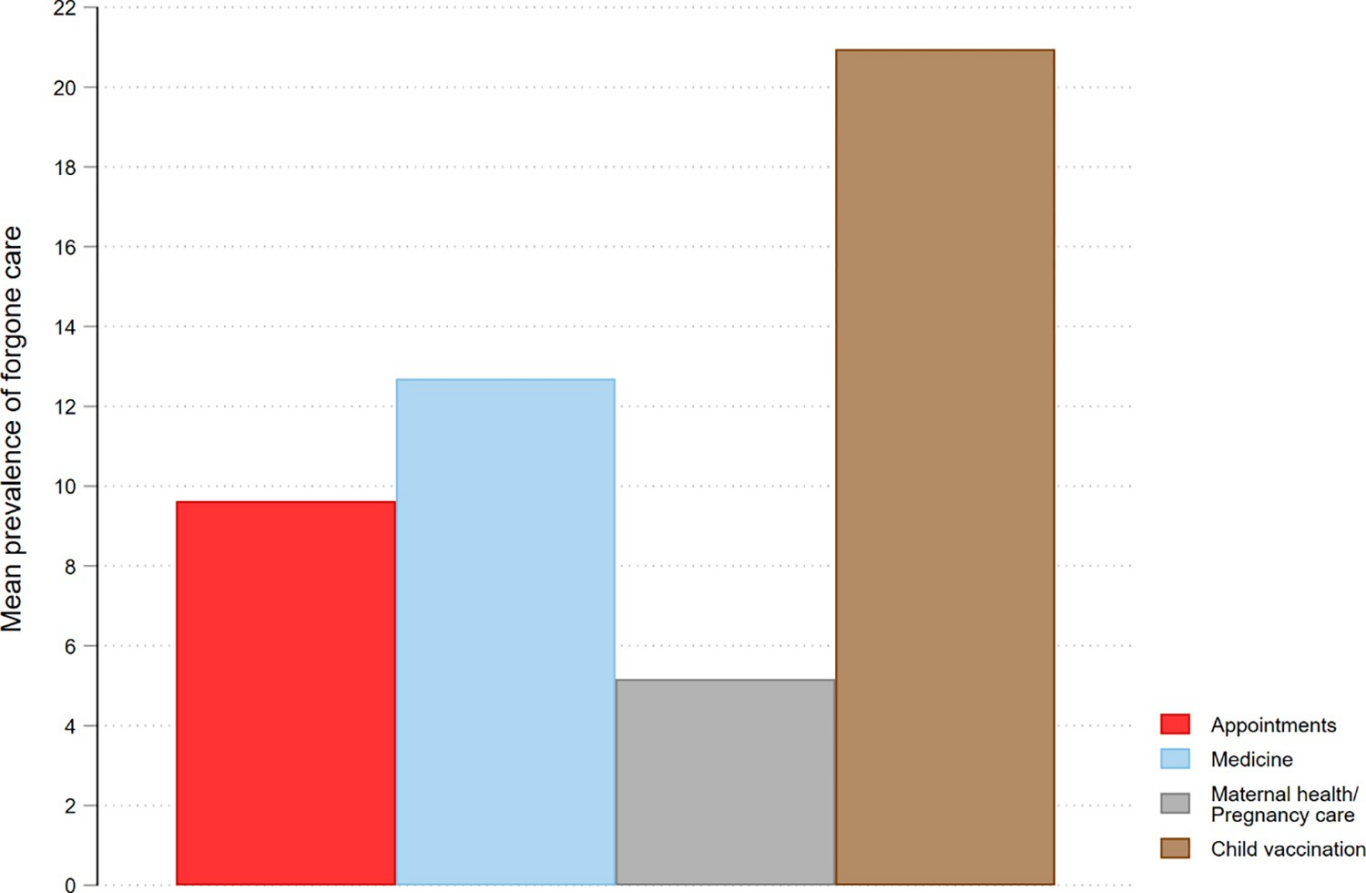
Prevalence of forgone care for four types of essential services during the early peri-outbreak period of the COVID-19 pandemic in Nigeria. *Notes*: authors’ calculations were based on weighted samples of Nigeria COVID-19 National longitudinal phone surveys (NLPS) 2020/2021 (rounds 1, 2, 3, 4, 9, 10 and 11) and 2021/2022 (rounds 1, 3,4 and 5).

Fig 5 and S2 Fig depict the decline in essential services sought during the early stages of the pandemic. The highest proportion of forgone care was observed for child vaccination (21.00%), followed by medicine (12.70%) and appointments (outpatient consultation) (9.60%). In contrast, the least forgone care was for maternal health/pregnancy care (5.20%). The trend for forgone care for these three essential services showed a similar reduction after the initial period, but with some variability, as shown in S2 Fig. Access to child vaccination, medicine, and maternal health/pregnancy care improved after January 2021, albeit with some interruptions between December 2021 and March 2022 for child vaccination and medicine, and between November and December 2021 for maternal/pregnancy care. As a result, the proportion of forgone care for maternal/pregnancy care reached its highest level at 14.40% during this period.

### Reasons for forgone care

**Fig 6:**
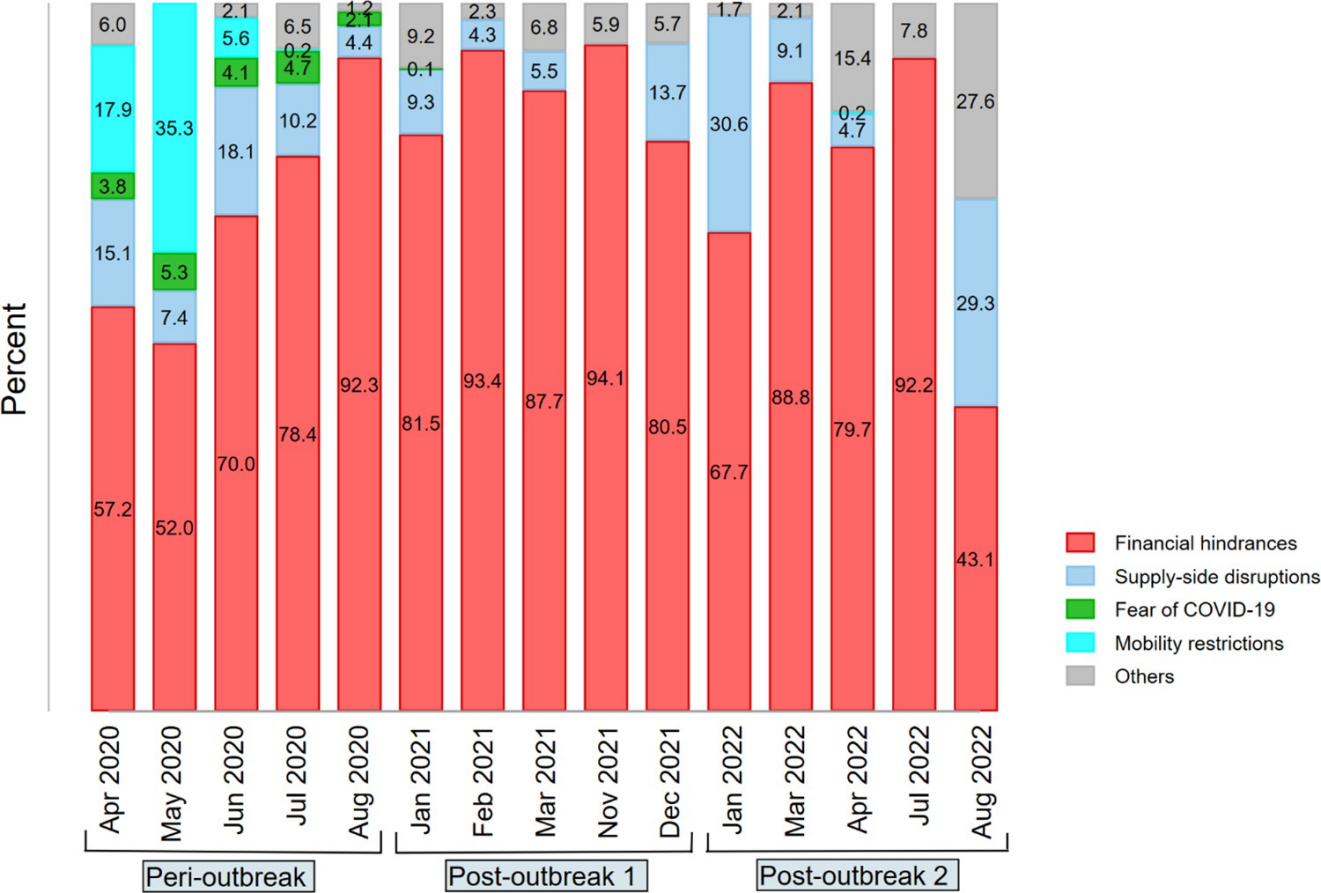
Variability in the reasons for forgone care during the different periods of the COVID-19 pandemic in Nigeria (2020–2022). *Notes*: The figure shows variability in the reasons households forgone care. Financial hindrances constitute the highest percentage throughout the pandemic. In the early pandemic, mobility restriction was next to financial hindrance, but was replaced by supply-side disruption later in the pandemic. The fear of COVID-19 constituted the least important reason for forgone care, especially beyond peri-outbreak period. Authors’ estimates were based on weighted samples of Nigeria’s COVID-19 National longitudinal phone surveys (NLPS) 2020/2021 (rounds 1, 2, 3, 4, 9, 10 and 11) and 2021/2022 (rounds 1, 3,4 and 5). *Peri-outbreak* represents a period from April to August 2020. *Post-outbreak 1* and *2* depict a period from January 2021 to December 2021, with some gaps from April to October 2021 and January to August 2022, respectively.

Fig 6 and S3 Fig show the reasons given by the households for forging medical care in general. Financial hindrances topped the list throughout the period examined, with a mean prevalence of 74.51% [95%CI: 69.71–78.79]. It continued to increase relative to other reasons after April/May 2020. Supply-side disruption was next to financial reasons (11.90% [95%CI: 8.98–15.61]) and persisted throughout the periods of the pandemic, more prominently in the post-outbreak 2. Mobility restriction was prominent in the early pandemic, ranking next to financial reasons with a prevalence of 23.77% [95%CI: 15.35–34.91] in April/May 2020; it became inconsequential after June 2020. The fear of COVID-19 constituted the least important reason for forgone care (with a mean prevalence of 5.66% [95%CI: 3.66–8.65] and ceased becoming an issue outside the peri-outbreak period. Supply-side and other reasons gradually displaced COVID-19 concerns and mobility restrictions in the post-outbreak periods with a mean prevalence of 19.41% [95%CI: 11.79–30.26] and 10.05% [95%CI: 4.34–21.60], respectively between January and August 2022.

The reasons cited for forgoing different types of essential services also varied (S4 Table). Except for child vaccination, financial hindrance was the most frequently cited reason for forgoing care, with prevalence of 48.56% [95%CI: 27.86–69.77] for maternal health/pregnancy care and 79.03% [95%CI: 67.96–87.01] for medicine. Supply-side disruption (43.09% [95%CI: 31.77–55.18]) and mobility restrictions (35.96% [95%CI: 26.23–46.99]) were the main reasons for households forgoing child vaccination.

### Determinants of household forgone care during the pandemic

Table 2 shows the results of the panel logistic regression for the determinants of household forgone care during the pandemic. Four household factors significantly impacted household forgone care during the lockdown period and throughout the pandemic. Household income loss significantly increased the odds of having forgone care by 2.23 [95%CI: 1.00–4.99], p<0.05 during the lockdown and 2.74 [95%CI: 1.45–5.17], p<0.01 for the whole duration of the pandemic compared with households with either stable or increased income. However, compared with households in the North-Central, households in the North-East were 75.00% (OR 0.25 [95%CI: 0.15–0.58]) and 72.00% (OR 0.28 [95%CI: 0.15–0.53]) significantly less likely (p<0.01) to experience forgone during the lockdown and the duration of the pandemic, respectively. Similarly, households in the North-West zones compared with North-Central were 70.00% (OR 0.30 [95%CI: 0.15–0.58]) and 64.00% (OR 0.36 [95%CI: 0.20–0.65]) significantly less likely (p<0.01) to experience forgone care during the lockdown and through the pandemic, respectively. Households with a married head compared with an unmarried/divorced household head were 70.00% (OR 0.30 [0.14–0.62]) and 69.00% (OR 0.31 [95%CI: 0.16–0.59]) significantly less likely (p<0.01) to experience forgone care during the lockdown and throughout the pandemic, respectively.

**Table 2:** Determinants of household vulnerability to forgone care during the pandemic in Nigeria (2020–2022) (Panel data logistic regression with random effect estimator).

Explanatory Variable	During the Pandemic Period	Lockdown Period Subsample^α^
	Odd Ratio	Odd Ratio
**Predisposing factors**		
Age of household head^β^	0.94 [0.88–1.02]	0.96 [0.88–1.05]
Age squared	1.00* [1.00–1.00]	1.00 [1.00–1.00]
Female household head	0.59 [0.28–1.26]	0.56 [0.25–1.24]
Married household head	0.31*** [0.16–0.59]	0.30*** [0.14–0.62]
Married female household head	6.07*** [1.72–21.47]	6.25* [0.97–40.27]
Literacy of household head	1.005 [0.63–1.61]	0.927 [0.57–1.51]
**Enabling (or disenabling) factors**		
Household size	0.98 [0.93–1.04]	0.96 [0.88–1.03]
Household with income loss^¶^	2.74*** [1.45–5.17]	2.23** [1.00–4.99]
Household that received assistance	0.73 [0.41–1.28]	0.41*** [0.21–0.77]
Job loss by respondent (Previously working)	1.87*** [1.25–2.79]	1.02 [0.68–1.52]
Poor household (pre-pandemic)^φ^	1.76** [1.12–2.75]	1.22 [0.76–1.96]
Household with food insecurity^§^	1.60** [1.12–2.31]	1.39 [0.88–2.19]
**Need factors**		
Household member with a disability (pre-pandemic)	1.34 [0.82–2.19]	1.36 [0.80–2.33]
NCD-affected household (pre-pandemic)	1.10 [0.66–1.81]	1.06 [0.61–1.85]
Household with an elderly member	0.77 [0.45–1.34]	0.83 [0.45–1.53]
**Environmental factors**		
Rural location	0.85 [0.54–1.32]	0.86 [0.53–1.42]
North-Central Zone	Reference	Reference
North-East Zone	0.28*** [0.15–0.53]	0.25*** [0.15–0.58]
North-West Zone	0.36*** [0.20–0.65]	0.30*** [0.15–0.58]
South-East Zone	1.04 [0.55–1.96]	1.01 [0.51–1.98]
South-South Zone	1.98** [1.09–3.58]	1.29 [0.63–2.62]
South-West Zone	0.84 [0.44–1.62]	0.54* [0.27–1.09]
Constant	0.58 [0.07–4.56]	2.17 [0.20–3.29]
Number of observations	1,335	638
Number of households	1,026	638

*Note*: Authors’ estimates were based on unweighted samples of Nigeria’s COVID-19 National longitudinal phone surveys (NLPS) 2020/2021 (rounds 1, 2, 3, 4, 9, 10 and 11) and 2021/2022 (rounds 1, 3,4 and 5).

^α^Regression performed on rounds 1 and 2 sub-sample, covering only the Lockdown period (April-June 2020).

^β^ Square of the age of household head included in the regression.

^¶^ Households with a reduction in income compared with the same month in the preceding year

^φ^ Households below the national poverty line pre-pandemic.

^§^ Measured by any adult that went without eating for a whole day in the past week.

*P values*: *** p<0.01

** p<0.05

* p<0.1

95% CIs are shown in square brackets.

Robust standard errors clustered at the enumeration area (EA).

Although not statistically significant, a household with a female head was 41% (OR 0.59 [95%CI: 0.28–1.26]) less likely to experience forgone care than a male-headed household. However, being a married female household head increased the odds of household forgone care by 6.07 [95%CI: 1.72–21.47], p<0.01 during the pandemic compared to the unmarried female household head.

For socioeconomic conditions, the odds of a household incurring a forgone care became significantly larger as the pandemic progressed. While households below the poverty line and those with food insecurity did not experience forgone care during the lockdown, they were 76% (OR 1.76 [95%CI: 1.12–2.75]) and 60% (OR 1.60 [95%CI: 1.12–2.31]), receptively more likely to experience forgone care (p< 0.05) as the pandemic progressed. This is similar for job loss with no significant impact on forgone care during the lockdown but was 87% (OR 1.87 [95%CI: 1.25–2.79]), p<0.01, more likely to induce forgone care as the pandemic progressed, compared with households without job loss. However, when compared with households without assistance, households that received assistance were 59% less likely to experience forgone care (OR 0.41 [95%CI: 0.21–0.77]), p<0.05, and this was only during the lockdown.

Apart from households in the South-South which had twice higher (OR 1.98 [95%CI: 1.09–3.58]), p<0.05 likelihood of inducing forgone care, compared with households in the North-Central, during the lockdown, the impact of the other geopolitical locations was lower during the entire length of the pandemic.

There was no evidence that a household head’s age or literacy level significantly affected household forgone care. Household size, rural-urban location, and having a household member with NCDs, disability, or old age also conferred no statistically significant impact on forgone care.

## Discussion

This study examined Nigeria’s prevalence and determinants of foregone essential care during the COVID-19 epidemic. In the early stages of the pandemic, over one-third of homes did not receive care. After the lockdown, this high prevalence of households forgoing care declined, but with an increase in the number of households requiring care. Financial constraints, mobility restrictions, and supply-side disruptions accounted for most forgone care. A similar trend and reasons were also observed for individual essential care category. Our findings suggest that household characteristics such as income loss, food insecurity, poverty, location in the South-South zone, and having a married female head increased households’ susceptibility to forgone care. In contrast, being a married head of a household, receiving support, and residing in the North-East or North-West zones were protective. Surprisingly, NCDs and disability did not affect forgone care.

The findings in our study align with the studies showing a high prevalence of forgone care during the early pandemic in other regions [11, 36]. A study in India reported that 23.4% of households experienced delayed/forgone care during early 2020 [36]. Tsuzaki and Taira (2022) also found the highest forgone care of 20.8% among Medicare beneficiaries in the summer of 2020 in the United States [37]. This study also confirmed progressive falls in the trend of forgone care during the pandemic. Other researchers have found a similar trend in forgone medical care during the pandemic’s later period after an initial rise in the early weeks [38, 39]. These findings show the considerable impact of the mobility restrictions on forgone care in the early pandemic. Unlike high income countries, the fear of COVID-19 infection did not affect forgone care in Nigeria [11, 40]. Low detection rates due to inadequate capacity for testing and disproportionately low fatality rates in Nigeria are possible explanations for this finding [9, 41]. Thus, rather than the COVID-19 infection, the restriction measures to control the pandemic escalated forgone care early in the pandemic.

Our study has demonstrated that childhood vaccination was the most affected during the pandemic. This aligns with numerous findings from various parts of the world [6], and is consistent with other studies which have shown significant declines in childhood vaccination coverage in Nigeria [42, 43]. The pandemic-induced mobility restrictions and healthcare disruption were the primary cause of this decline, as evidenced by the fact that hard-to-reach rural and security-prone areas were most affected [42–44]. This has serious implications for the fight against vaccine-preventable diseases, particularly in the most disadvantaged areas of the country.

Our study also revealed that maternal health/pregnancy care, essential medicine and routine appointments, were also affected with forgone care. The latter two which were markedly affected are crucial for the management of NCDs and other chronic diseases [45, 46]. Our findings are consistent with studies which have shown disruptions in maternal health services, including family planning [15, 47, 48], an increase in missed appointments [45, 49, 50], and a lack of access to essential medicines in Nigeria [4, 13]. The general shortfall in the supply of essential medicines, and concomitant increase in prices and strain socioeconomic conditions explain these [13, 46, 51]. Maternal health services were the least affected by the pandemic and this relative resilience could be attributed innovation and adaptation in the place, time and mode of service delivery aimed at enhancing patient access and control, technically supported by international organizations [52].

Kakietek et al. (2022) corroborate our findings, showing that financial constraints were the most frequently reported reason for forgone care in Nigeria and LMICs [11]. Similarly, financial vulnerability has been demonstrated to substantially limit access to care during the pandemic [53, 54]. Iliyasu et al. (2023) contradicted our study, indicating that the fear of COVID-19 was the most important reason for not accessing maternal care during lockdown. While our study is nationally representative, this study was conducted in a rural setting with only 389 participants [55]. However, the spike in the prevalence of forgone care that we observed in late November 2021 can be attributed to the fear of the new Omicro variants and the corresponding increase in cases during that period [56].

A substantial household pre-pandemic financial susceptibility is plausible, with about 40% of Nigerians below poverty and high catastrophic OOP health spending [41, 42]. High OOP has been associated with increased forgone care during the pandemic in Europe [57]. Using propensity score matching, a study in India found that health insurance coverage significantly increased healthcare utilization during the pandemic [58]. Another study from 195 countries employed a difference-in-differences analysis to show that countries with higher universal health coverage had greater ability to provide essential health services and minimise disruptions to service delivery during the pandemic [59]. Also, some pandemic-induced factors, such as income reduction, job loss and food insecurity, as reported in our study and others [10] and reported escalation in inflation and healthcare costs [60, 61], must have contributed to the financial hardship and the resultant economic-driven forgone care in Nigeria during the pandemic.

Other socioeconomic characteristics that increased household susceptibility to financial difficulties, such as loss of income or job and food insecurity, were also associated with a greater likelihood of forgoing care [62–64]. Consistent with our findings, studies in other countries have shown social support, such as palliatives and monetary transfers, increased access to care during the epidemic [65, 66].

Although the prevalence of forgone care dropped after the lockdown, the large volume of households requiring care this time, which is similar to Hategeka et al. (2021) findings in the Democratic Republic of Congo (DRC), and the rising COVID-19 cases and its sequelae cumulatively impacted the capacity of the fragile and battered Nigeria healthcare system to maintain high-quality care after the lockdown [23, 67, 68]. The drop in needed care during the lockdown from the pre-pandemic level most likely reflected reductions in infectious diseases owing to COVID-19 preventive measures like hand washing and social distancing and reductions in road traffic accidents due to mobility restrictions. The rise in pent-up demands for missed non-COVID-19 care during the lockdown was most likely responsible for the spillover afterwards.

Our findings are consistent with studies showing massive disruption in the supply of health services and its negative impact on forgone care during the pandemic [38, 69]. A WHO report revealed that the interruption of essential care continued in many countries long after the pandemic [70]. Shapira et al. (2021) showed a persistent disruption in the continuity of maternal and child healthcare (MCHC) in Nigeria [23]. During the lockdown in Nigeria, restrictions of movement, fear of contracting COVID-19 infection at work and poor working conditions were likely disruptors of essential care [71]. Afterwards, factors such as rescheduling of appointments, redeployment of staff to COVID-19 care, and insufficient medical equipment and supplies, complicated by a poorly-funded and ill-prepared healthcare system, emanating from high-volume care, escalated the situation [5].

The marital status of the household head seems to lower the possibility of forgone care because marriage provides an avenue for additional emotional and financial support from partners. However, when the married household head was a female, the probability of forgone care increased considerably. A study has associated married women with avoidance of care during the pandemic [72]. This avoidance might be because of additional responsibilities such as caregiving and housekeeping that might hinder their financial independence and health-seeking behaviour. They may also have fewer social and emotional support networks, affecting their ability to navigate the healthcare system and make health-seeking decisions. Additionally, women were less likely to earn more than men, particularly during the pandemic [73].

The geographical location of a household appears to impact forgone care. While living in the South-South increased the odds of forgone care, contrary to expectations, we found that living in the North-East and North-West zones minimised the probability of forgone care. These are three zones ravaged by arms conflicts in Nigeria [74]. A report has confirmed a substantial decrease in service disruption in the internally-displaced persons (IDP) camps in the North East [75]. The situation in the North-East and North-West zones might not be unconnected with improved humanitarian efforts in these areas during the pandemic.

While chronic conditions such as NCDs, disability and old age have been associated with an increased prevalence of forgone care [76–78], our study showed no statistically significant impact. This might indicate the priority households placed on the care of chronic conditions, considering Nigeria’s relatively lower COVID-19 fatality compared to countries where the other studies were conducted [9].

Some limitations relating to data collection were likely to introduce biases in our study. First, phone surveys usually exclude a portion of the population lacking access to phones and stable networks [79]. This selection bias was insignificant since most households had attached phone numbers, and the interview response rate, except for the baseline survey, was above 90%. We corrected for possible biases using sampling weight in our estimates. Second, individual-level estimates are likely skewed due to the interviewees’ disproportionate likelihood of being male [80]. Our calculations were at the household level so that this gender-specific bias could only have negligible effects. Third, the data supplied during the survey were self-reported, which could introduce recall bias. The short recall periods used in the survey would have reduced the possibility of such bias. Lastly, the different data collection methods for pre-pandemic GHSP and the COVID-19 NLPS were unlikely to impact significantly on our result because our analysis relied heavily only on the NLPS.

## Conclusion

Our study expanded the literature on access to healthcare during the COVID-19 pandemic, demonstrating that the pandemic widened the pre-existing inequalities in health access. The study underpins the need for policy strengthening social protection systems and financial risk protection to alleviate the impact of household socioeconomic vulnerability. The capacity and resilience of the health system must also be strengthened to mitigate ensuing disruptions in service provision. Moreover, developing policy frameworks fostering teleconsultations and balancing lockdown benefits and healthcare accessibility should be prioritised during future pandemics. However, further work is recommended to quantify the exact impacts of the lockdown and the perceived risk of COVID-19 on forgone care in Nigeria.

## Supporting information

S1 Table The distribution of respondents across the seven rounds of the national longitudinal phone survey (phase 1) 2020/2021 used. (DOCX)

S2 Table The distribution of respondents across the three rounds of the national longitudinal phone survey (phase 2) 2021/222 used. (DOCX)

S3 Table Prevalence of forgone care and population affected during the three-point periods of the pandemic in Nigeria. (DOCX)

S4 Table Reasons for forgone care for different essential care services during early COVID-19 pandemic in Nigeria. (DOCX)

S1 Fig Time-Trend Plots of Forgone Care (Upper Panel) Compared to the Number of COVID-19 Cases and Deaths and Stringency Index (Lower Panel) Across Time During the Pandemic in Nigeria (2020–2022).
(TIF)

S2 Fig **(a-c)**: Evolution in the prevalence of medicine, maternal health/pregnancy care and child vaccination services during the COVID-19 pandemic in Nigeria.
(TIF)

S3 Fig Overall prevalence of the reasons for forgone care during the COVID-19 pandemic in Nigeria. (TIF)
