# Supplementary material for: The evolution of household forgone essential care and its determinants during the COVID-19 pandemic in Nigeria: A longitudinal analysis
Source: PLoS One. 2024 Apr 2;19(4):e0296301. doi: 10.1371/journal.pone.0296301 (PMC10986961; doi:10.1371/journal.pone.0296301)
Supplement: S1 Table — (DOCX) [file pone.0296301.s001.docx]

***Table S1*: The distribution of respondents across the seven rounds of the National Longitudinal Phone Survey (phase 1) 2020/2021 used**

| **Survey round** | **Period of interview** | **Number of households attempted** | **Number of households successfully contacted** | **Number of households successfully interviewed** | **Response rate** |
| --- | --- | --- | --- | --- | --- |
| 1^st^ round (Baseline) | April-May 2020 | 3,000 | 2,070 | 1,950 | 65.0% |
| 2^nd^ round | June 2020 | 1,950 | 1,852 | 1,820 | 93.3% |
| 3^rd^ round | July 2020 | 1,925 | 1,837 | 1,790 | 93.0% |
| 4^th^ round | August 2020 | 1,881 | 1,819 | 1,789 | 95.1% |
| 9^th^ round | January 2021 | 1,789 | 1,712 | 1,706 | 95.4% |
| 10^th^ round | February 2021 | 1,785 | 1,716 | 1,699 | 95.2% |
| 11^th^ round | March 2021 | 1,777 | 1,695 | 1,680 | 94.5% |
