# Supplementary material for: The evolution of household forgone essential care and its determinants during the COVID-19 pandemic in Nigeria: A longitudinal analysis
Source: PLoS One. 2024 Apr 2;19(4):e0296301. doi: 10.1371/journal.pone.0296301 (PMC10986961; doi:10.1371/journal.pone.0296301)
Supplement: S2 Table — (DOCX) [file pone.0296301.s002.docx]

***Table S2*: The distribution of respondents across the three rounds of the National Longitudinal Phone Survey (phase 2) 2021/222 used**

| **Survey round** | **Period of interview** | **Number of households attempted** | **Number of households successfully contacted** | **Number of households successfully interviewed** | **Response rate** |
| --- | --- | --- | --- | --- | --- |
| 1^st^ round (Baseline) | November 2021-January 2022 | 4,440 | 3,132 | 2,922 | 65.8% |
| 3^rd^ round | March-April 2022 | 2,881 | 2,694 | 2,647 | 91.9% |
| 4^th^ round | June 2022 | 2,852 | 2,646 | 2,605 | 91.3% |
| 5^th^ round | July-Aug 2022 | 2,824 | 2,610 | 2,574 | 91.1% |
