# Supplementary material for: The evolution of household forgone essential care and its determinants during the COVID-19 pandemic in Nigeria: A longitudinal analysis
Source: PLoS One. 2024 Apr 2;19(4):e0296301. doi: 10.1371/journal.pone.0296301 (PMC10986961; doi:10.1371/journal.pone.0296301)
Supplement: S3 Table — (DOCX) [file pone.0296301.s003.docx]

***Table S3*: Prevalence of forgone care and population affected during the three-point periods of the pandemic in Nigeria**

| **Forgone care** | **Peri-outbreak** | **Post-outbreak 1** | **Post-outbreak 2** |
| --- | --- | --- | --- |
| Mean prevalence (%) | 15.56  [13.49–17.63] | 7.38  [6.09–8.66] | 2.63  [1.71– 3.56] |
| Number of households affected | 6307634  [5468084–7147184] | 3789602  [3130424–4448780] | 1045058  [678186.6–1411928] |
| *Notes:* Authors’ calculations were based on weighted samples of Nigeria COVID-19 National longitudinal phone surveys (NLPS) 2020/2021 (rounds 1, 2, 3, 4, 9, 10 and 11) and 2021/2022 (rounds 1, 3,4 and 5).  *Peri-outbreak* represents a period from April to August 2020. *Post-outbreak 1 and 2* represent a period from January 2021 to December 2021, with some gaps from April to October 2021 and January to August 2022, respectively.  95% CIs are shown in square brackets. | | | |
