# Supplementary material for: The evolution of household forgone essential care and its determinants during the COVID-19 pandemic in Nigeria: A longitudinal analysis
Source: PLoS One. 2024 Apr 2;19(4):e0296301. doi: 10.1371/journal.pone.0296301 (PMC10986961; doi:10.1371/journal.pone.0296301)
Supplement: S4 Table — (DOCX) [file pone.0296301.s004.docx]

***Table S4*: Reasons for forgone care for different essential care services during early COVID-19 pandemic in Nigeria**

| **Type of service** | **Reason for forgone care** | | | | |
| --- | --- | --- | --- | --- | --- |
|  | **Financial hindrance** | **Supply-side disruption** | **Fear of COVID-19** | **Mobility restriction** | **Others** |
| Medicine *n (%)* | 105 (79.03) [67.96–87.01] | 15 (9.84) [5.03–18.38] | - | 16 (11.13) [5.43–21.45] | - |
| Maternal health/Pregnancy care *n (%)* | 19 (48.56) [27.86–69.77] | 10 (17.30) [7.14–36.24] | 1(0.34) [0.04–2.69] | 3 (11.98) [2.43–42.66] | 6 (21.83) [7.64–48.53] |
| Child vaccination *n (%)* | 20 (11.98) [6.92–19.95] | 55 (43.09) [31.77–55.18] | 10 (4.62) [2.25–9.23] | 57 (35.96) [26.23–46.99] | 8 (4.36) [1.33–13.33] |
| *Notes:* Authors’ calculations were based on weighted samples of Nigeria COVID-19 National longitudinal phone surveys (NLPS) 2020/2021 (rounds 1, 2, 3, 4, 9, 10 and 11) and 2021/2022 (rounds 1, 3, 4 and 5).  95% CIs are shown in square brackets. | | | | | |
