# Supplementary figures and images for: The evolution of household forgone essential care and its determinants during the COVID-19 pandemic in Nigeria: A longitudinal analysis
Source: PLoS One. 2024 Apr 2;19(4):e0296301. doi: 10.1371/journal.pone.0296301 (PMC10986961; doi:10.1371/journal.pone.0296301)

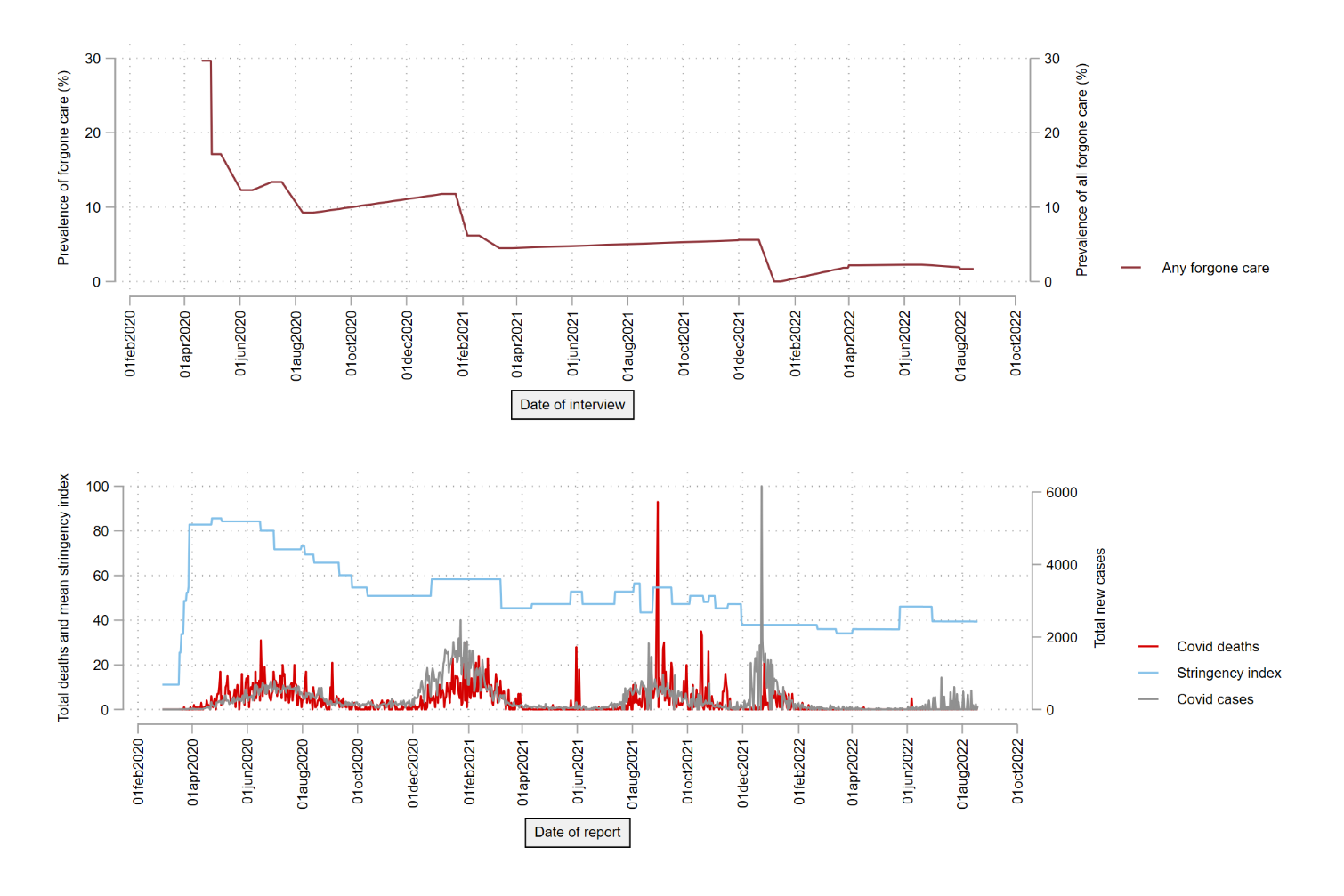

Supplement: S1 Fig — Time-Trend Plots of Forgone Care (Upper Panel) Compared to the Number of COVID-19 Cases and Deaths and Stringency Index (Lower Panel) Across Time During the Pandemic in Nigeria (2020–2022). (TIF) [file pone.0296301.s005.tif]

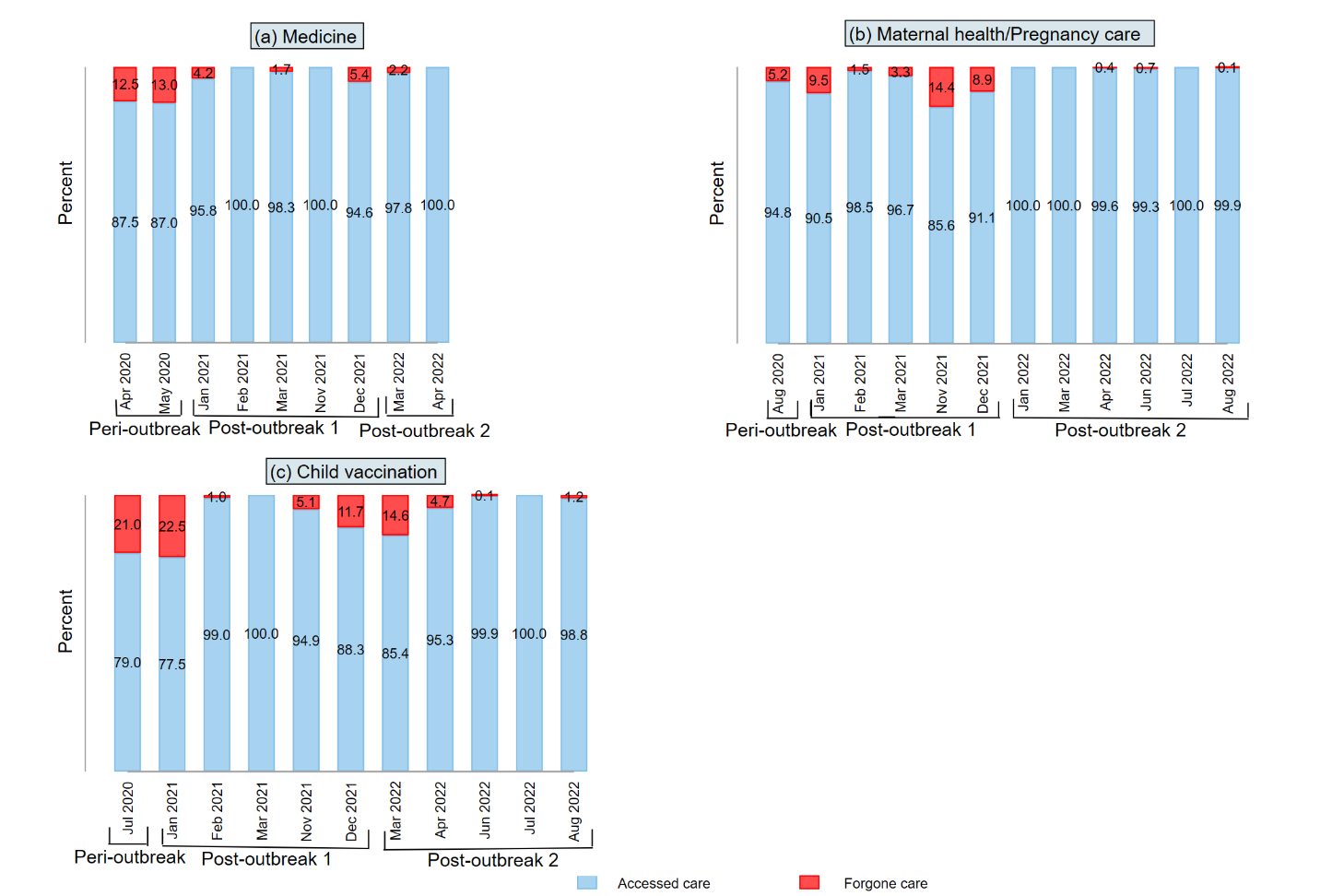

Supplement: S2 Fig — (a-c): Evolution in the prevalence of medicine, maternal health/pregnancy care and child vaccination services during the COVID-19 pandemic in Nigeria. (TIF) [file pone.0296301.s006.tif]

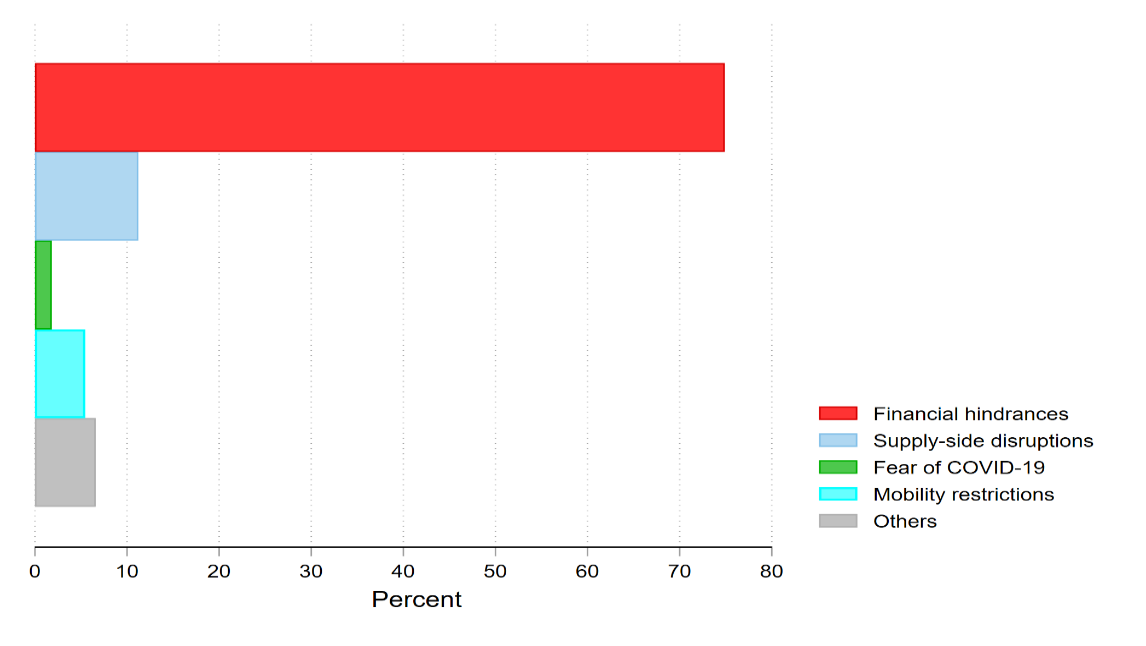

Supplement: S3 Fig — (TIF) [file pone.0296301.s007.tif]
